# Identification of common genetic variants in *KCNQ* family genes associated with gastric cancer survival in a Chinese population

**DOI:** 10.7555/JBR.38.20240040

**Published:** 2024-05-29

**Authors:** Yuetong Chen, Chen Li, Yi Shi, Jiali Dai, Yixuan Meng, Shuwei Li, Cuiju Tang, Dongying Gu, Jinfei Chen

**Affiliations:** 1 Department of Radiation Oncology, the Affiliated Suzhou Hospital of Nanjing Medical University, Suzhou Municipal Hospital, Gusu School, Nanjing Medical University, Suzhou, Jiangsu 215008, China; 2 Department of Oncology, Nanjing First Hospital, Nanjing Medical University, Nanjing, Jiangsu 210006, China; 3 Department of Oncology, the First Affiliated Hospital of Wenzhou Medical University, Wenzhou, Zhejiang 325015, China; 4 Department of Oncology, the First Affiliated Hospital of Nanjing Medical University, Nanjing, Jiangsu 210029, China; 5 Department of Environmental Genomics, Jiangsu Key Laboratory of Cancer Biomarkers, Prevention and Treatment, Collaborative Innovation Center for Cancer Personalized Medicine, Nanjing Medical University, Nanjing, Jiangsu 211166, China; 6 Department of Genetic Toxicology, the Key Laboratory of Modern Toxicology of Ministry of Education, Center for Global Health, School of Public Health, Nanjing Medical University, Nanjing, Jiangsu 211166, China

**Keywords:** gastric cancer, survival, genetic variants, ionic channels

## Abstract

The *KCNQ* family of genes (*KCNQ1*–*KCNQ5*), encoding voltage-gated K^+^ (Kv) channels, have been demonstrated to play potential pathophysiological roles in cancers. However, the associations between genetic variants located in *KCNQ* family genes and gastric cancer survival remain unclear. In this study, a large-scale cohort comprising 1135 Chinese gastric cancer patients was enrolled to identify genetic variants in *KCNQ* family genes associated with overall survival (OS). Based on the survival evaluation of all five *KCNQ* family genes, *KCNQ1* was selected for subsequent genetic analysis. In both Cox regression model and stepwise Cox regression model used to evaluate survival-related genetic variants, we found that *KCNQ1* rs10832417G>T was associated with an increased OS in gastric cancer patients (adjusted hazards ratio [HR] = 0.84, 95% confidence interval [CI]: 0.72–0.98, *P* = 0.023). Subsequently, a nomogram was constructed to enhance the prognostic capacity and clinical translation of rs10832417 variants. The rs10832417 T allele was predicted to increase the minimum free energy of the secondary structure. Furthermore, we observed that gastric cancer patients with downregulated *KCNQ1* expression had a poorer survival across multiple public datasets. The findings of the present study indicate that *KCNQ1* rs10832417 may serve as an independent prognostic predictor of gastric cancer, providing novel insights into the progression and survival of the disease.

## Introduction

Gastric cancer ranks the fifth in malignancy incidence and the fourth in cancer-related deaths worldwide, with the highest incidence and mortality rates found in Eastern Asia^[1–2]^. Because of the asymptomatic nature of its early stages, gastric cancer is often diagnosed at advanced stages, leaving patients with limited treatment options and a poor prognosis^[3]^. Currently, the management of gastric cancer is still mainly based on the tumor-node-metastasis (TNM) system, which ignores intertumoral and interpatient heterogeneity and may result in overtreatment or insufficient treatment^[4]^. Thus, it is urgent to identify robust molecular biomarkers to guide therapeutic decisions and improve the quality of life of gastric cancer patients.

Voltage-gated K^+^ (Kv) channels are key regulators of various functional activities in numerous cell types and are encoded by over 70 genes in the human genome. Among these, the Kv7 channel family consists of five members (Kv7.1–Kv7.5). Compared with other Kv channel families, the Kv7 channel family is characterized by relatively negative voltage dependence, little or no inactivation, and slow activation processes, which contribute to their important roles in cellular physiology^[5]^. Different isoforms of the Kv7 channel family, ranging from 650 to 940 amino acids in length, are encoded by *KCNQ* family genes (*KCNQ1*–*KCNQ5*) located at chromosomal loci *11p15*, *20q13*, *8q24*, *1p34*, and *6q13*, respectively^[6]^. All five proteins consist of six transmembrane domains, a pore loop (P-loop, P), a short N-terminus, and a long C-terminus, which have extremely high homologous transmembrane regions, sharing approximately 30%–65% amino acid identity^[7–8]^. The fourth segment of the transmembrane domain is commonly responsible for voltage sensing and is contributed by six positively charged amino acids, except for *KCNQ1*, which has only four amino acids^[8]^. In addition, the C-terminus plays a crucial role in the subunit assembly and processes many functional mutations and variants^[5]^.

Extensive investigations on *KCNQ* family genes have revealed their different physiological functions in different tissues, disease progression and treatment. It was reported that the development and metastasis of renal cell carcinoma were promoted by the miR-140-5p/*KLF9*/*KCNQ1* axis, revealing a novel pathogenic mechanism underlying renal cell carcinoma^[9]^. Downregulation of *KCNQ2* was reported to be correlated with oxaliplatin-induced trigeminal neuropathic pain that occurs in the majority of advanced colorectal cancer patients receiving oxaliplatin-based chemotherapy, and the potentiator retigabine of KCNQ2 alleviated neuropathic pain in rat models^[10]^. The activation of KCNQ2/3 channels induced the apoptosis of neuronal cells, suggesting their important roles in the regulation of neuronal cell viability^[11]^.

Moreover, studies focusing on genetic variants located in *KCNQ* family genes have improved our understanding of the mechanisms underlying both the occurrence and treatment of human diseases over the past decades. For example, *KCNQ1* rs2237892 was found to be associated with infant postnatal rapid weight gain^[12]^, and contributed to the risk of type 2 diabetes mellitus and its related complications^[13]^. Another genome-wide association study reported that rs9351963 in *KCNQ5* might act as a predictor for diarrhea in irinotecan-treated cancer patients^[14]^. Notably, single nucleotide polymorphisms (SNPs) in *KCNQ* family genes may have different biological effects on diseases in different populations. For example, the rs34287852 variant in *KCNQ4* was reported to have the opposite susceptibility influence on noise-induced hearing loss in Swedish and Polish populations^[15–16]^.

In the present study, we evaluated the associations between candidate SNPs in *KCNQ* family genes and survival in a large clinical cohort of a Chinese gastric cancer population, further assessed the prognostic performance of the selected SNPs visualized by a nomogram, and investigated their potential biological functions in multiple datasets.

## Subjects and methods

### Study participants

In the present study, a total of 1135 gastric cancer patients^[17–18]^ of Chinese descent were enrolled at Nanjing First Hospital of Nanjing Medical University between 2005 and 2012, with no age or sex restrictions (***Supplementary Table 1***, available online). All patients were pathologically diagnosed with primary gastric cancer after surgery, and also were confirmed by electronic medical records. Patients with a history of any cancer prior to the present study were excluded. Patients who received chemotherapy or radiotherapy were also excluded. Follow-ups were conducted *via* telephone calls and reviews of electronic medical records, with a maximum follow-up time of 105 months and a median follow-up time of 31 months. Overall survival (OS) was set as the primary endpoint of the present study and defined as the duration from the date of surgery to death or the last follow-up date. All patients signed a written informed consent before recruitment to the present study. The study approval was obtained from the Institutional Review Board of Nanjing Medical University. All procedures were performed according to the Helsinki Declaration.

### SNP genotyping

Formalin-fixed paraffin-embedded (FFPE) tissue samples were used to extract genomic DNA using the TGuide FFPE DNA Extraction Kit (TIANGEN, Beijing, China). The concentration and purity of all DNA samples were evaluated by a Nanodrop 2000 spectrophotometer to ensure they met the qualification for genotyping. The TaqMan genotyping assay of candidate SNPs was conducted by an ABI 7900HT real-time PCR system (Applied Biosystems, Foster City, CA, USA). Subjects that failed to reach a genotype call rate of 95% were excluded. The assessment of genotyping consistency was based on 10% of randomly selected samples using the same assay system, with a concordance rate of 100%. For quality control, genotype analysis was performed by two studies in a double-blinded manner.

### SNP selection and quality control

**Figure 1:**
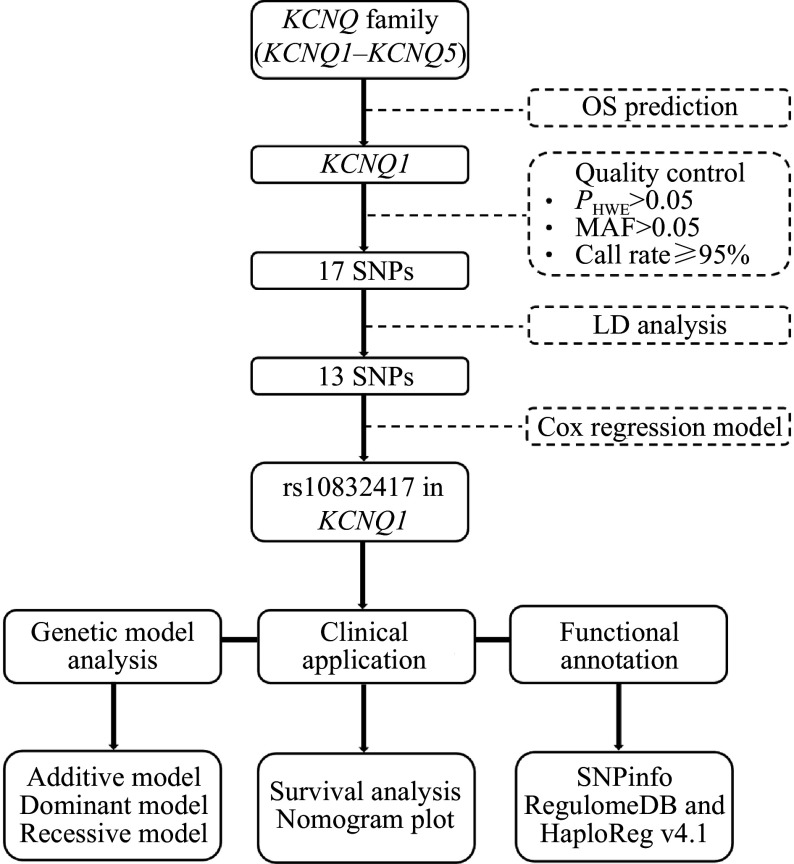
Diagram for SNP selection in *KCNQ* family genes. Abbreviations: OS, overall survival; HWE, Hardy-Weinberg equilibrium; MAF, minor allele frequency; SNP, single nucleotide polymorphism; LD, linkage disequilibrium.

We downloaded the analysis results for OS divided by the first and third quartiles of *KCNQ1*–*5* expression levels in The Cancer Genome Atlas (TCGA)-stomach adenocarcinoma (STAD) dataset from GEPIA2 (http://gepia2.cancer-pku.cn/#index). As shown in ***Supplementary Fig. 1*** (available online), only *KCNQ1* remained significant in the OS analysis. A total of 26 SNPs located in the *KCNQ1* gene were used for further analysis (***Supplementary Table 2***, available online). The following criteria were used for quality control of the abovementioned SNPs based on the Chinese Han Beijing (CHB) and Japanese in Tokyo (JPT) data from the 1000 Genomes Project Phase 3: (a) *P*-value of Hardy-Weinberg equilibrium > 0.05 and (b) minor allele frequency > 0.05. In addition, candidate SNPs with a genotyping success rate of less than 95% in our in-house cohort were excluded (***Supplementary Table 2***). A total of 17 SNPs remained for the next linkage disequilibrium (LD) analysis using an online tool developed by the National Cancer Institute (https://ldlink.nih.gov) based on the CHB and JPT datasets from the 1000 Genomes Project Phase 3 (*r*^*2*^ ≥ 0.6) (***Supplementary Fig. 2***, available online). Finally, only 13 SNPs (*i.e.*, rs7108478, rs7942590, rs2106464, rs11023485, rs11023535, rs10832417, rs12573965, rs2075868, rs12271234, rs10832514, rs16928527, rs61870802, and rs2283194) were included for survival analysis in our clinical cohort. The detailed selection process is shown in ***Fig. 1***.

### Functional annotation

SNPinfo (https://snpinfo.niehs.nih.gov/), RegulomeDB (https://www.regulomedb.org/regulome-search), and HaploReg v4.1 (https://pubs.broadinstitute.org/mammals/haploreg/haploreg.php) were used for the functional annotation of the candidate SNPs. The effects of secondary structures induced by different SNP genotypes were predicted using the RNAfold web server (http://rna.tbi.univie.ac.at/). Moreover, several online bioinformatic web portals were used to investigate the potential biological mechanisms of *KCNQ1* in both gastric cancer and pan-cancers. Protein-protein interaction networks of *KCNQ1* were assessed by the Search Tool for the Retrieval of Interacting Genes/Proteins (STRING) (https://string-db.org/). Different expression levels of *KCNQ1* among normal, gastric cancer, and pan-cancer tissues were comprehensively estimated through GTEx (https://www.gtexportal.org/), GEPIA2, and TISIDB (http://cis.hku.hk/TISIDB/index.php). We used the data from both TCGA-STAD in GEPIA2 and several Gene Expression Omnibus (GEO) datasets in Kaplan-Meier Plotter (https://kmplot.com/analysis/index.php) to assess the prognostic potential of *KCNQ1*. Multiple gastric cancer datasets from TIMER 2.0 (http://timer.cistrome.org/) and cBioPortal for cancer genomics (https://www.cbioportal.org/) were used to investigate the gene mutation status of *KCNQ1*.

### Statistical analysis

Statistical analysis in the present study was performed using Plink (version 1.9, http://zzz.bwh.harvard.edu/plink/) and R (version 4.0.5, https://www.r-project.org/). Multivariable Cox regression models under additive genetic models were used to estimate the associations between each SNP and survival time, adjusted for age, sex, smoking status, and drinking status. The best candidate factors for survival of patients with gastric cancer were selected by stepwise Cox regression analysis, with *P* < 0.05 for entry and *P* > 0.10 for removal. Survival analysis was assessed by the Kaplan-Meier method, and survival comparisons among groups were calculated by the log-rank test. A nomogram was constructed based on Cox regression models to visualize the survival probability of gastric cancer patients with different genotypes, and the C-index was calculated to evaluate the discrimination efficiency of each model. Calibration plots were used to present the results of bootstrap resampling validations in the internal cohort. *P* < 0.05 was considered statistically significant for all tests.

## Results

### Demographic and clinical characteristics of the study population

The baseline characteristics of our gastric cancer cohort are shown in ***Supplementary Table 1***. In brief, a total of 1135 gastric cancer patients were enrolled in the present study, including 433 mortality cases at the primary endpoint. In the demographic and clinicopathologic subgroups, we observed worse OS outcomes in patients with older age, drinking status, larger tumor size, poorer TNM classification (including depth of invasion, lymph node metastasis, distant metastasis, and TNM stage), and the diffuse subtype (*P* < 0.05).

### Identification of independent SNPs associated with gastric cancer survival

The detailed process of the whole study is presented in ***Fig. 1***. We selected *KCNQ 1* as the candidate gene for further study because of its specific survival significance among *KCNQ1*–*KCNQ5* based on the TCGA-STAD dataset. Then, we performed the quality control and further LD analysis of candidate SNPs based on the CHB and JPT data from the 1000 Genomes Project Phase 3.

Multivariable Cox regression models were applied to determine the contribution of candidate SNPs to OS in gastric cancer patients, with adjustments for age, sex, smoking status, and drinking status. The results showed that only two variants (rs2106464 and rs10832417) demonstrated significant associations with OS, at *P* < 0.05 under additive models (***Supplementary Table 2***). In addition, through stratification analysis of their different genotypes, we found that the T allele was associated with a better survival in both rs2106464 (adjusted hazards ratio [HR] = 0.75, 95% confidence interval [CI]: 0.58–0.96, *P* = 0.022 in an additive model; adjusted HR = 0.74, 95% CI: 0.57–0.96, *P* = 0.025 in a dominant model) and rs10832417 (adjusted HR = 0.84, 95% CI: 0.72–0.98, *P* = 0.023 in an additive model; adjusted HR = 0.76, 95% CI: 0.63–0.91, *P* = 0.004 in a dominant model) (***Table 1***).

**Table 1:** Associations of rs2106464 and rs10832417 with survival of gastric cancer patients

SNPs	Genotype	Patient (*n*)	Death (*n*)	HR (95% CI)^a^	*P* ^a^
rs2106464	CC	929	365	1	
	CT	192	64	0.75 (0.57, 0.98)	**0.033**
	TT	7	2	0.54 (0.13, 2.16)	0.383
	Additive model			0.75 (0.58, 0.96)	**0.022**
	Dominant model			0.74 (0.57, 0.96)	**0.025**
	Recessive model			0.56 (0.14, 2.26)	0.420
rs10832417	GG	581	242	1	
	GT	459	156	0.73 (0.60, 0.90)	**0.003**
	TT	92	35	0.87 (0.61, 1.25)	0.459
	Additive model			0.84 (0.72, 0.98)	**0.023**
	Dominant model			0.76 (0.63, 0.91)	**0.004**
	Recessive model			1.00 (0.71, 1.41)	0.993
^a^Adjusted for age, sex, smoking, and drinking status. Bold font indicates *P*-value < 0.05. Abbreviations: SNP, single nucleotide polymorphism; HR, hazards ratio; CI, confidence interval.					

We therefore performed the stepwise multivariable Cox regression analysis to identify independent predictors of gastric cancer OS. The analysis included dominant genetic models of the two aforementioned SNPs, age, sex, smoking status, drinking status, and clinical variables (tumor site, tumor size, TNM stage, and Lauren type). Finally, as shown in ***Table 2***, rs10832417 was found to be an independent predictor of gastric cancer survival (HR = 0.77, 95% CI: 0.63–0.93, *P* = 0.007). Taken together, these results based on a large population provide solid statistical evidence that *KCNQ1* rs10832417G>T may be an independent protector of gastric cancer survival.

**Table 2:** Stepwise Cox regression analysis of the overall survival of gastric cancer patients

Variables	*β*	SE	HR (95% CI)	*P*
Age (≥60 years *vs.* <60 years)	0.43	0.10	1.54 (1.26, 1.89)	<0.001
Drinking status (yes *vs.* no)	0.40	0.16	1.50 (1.09, 2.06)	0.013
Tumor size (>5 cm *vs.* ≤5 cm)	0.31	0.10	1.36 (1.12, 1.65)	0.002
TNM stage (Ⅲ/Ⅳ *vs.* Ⅰ/Ⅱ)	1.05	0.13	2.86 (2.21, 3.70)	<0.001
Lauren type (diffuse/others *vs.* intestinal)	0.31	0.10	1.36 (1.11, 1.67)	0.003
rs10832417 (GT/TT *vs.* GG)	−0.26	0.10	0.77 (0.63, 0.93)	0.007
Abbreviations: SE, standard error; HR, hazards ratio; CI, confidence interval.				

### Identification of rs10832417 genetic effects on gastric cancer survival

**Figure 2:**
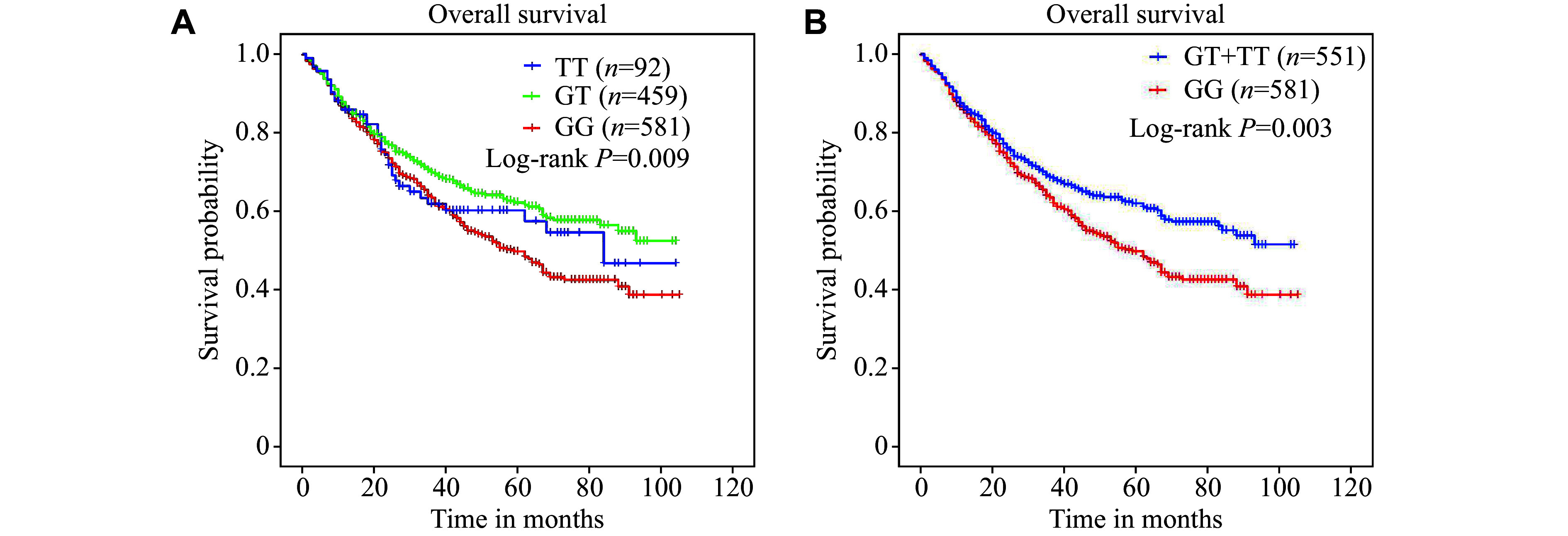
Kaplan-Meier analysis of rs10832417 genotypes for overall survival in gastric cancer patients. A and B: Kaplan-Meier curves of overall survival stratified by different genotypes of rs10832417 in our in-house gastric cancer cohort.

Kaplan-Meier survival curves were used to investigate genetic effects of rs10832417 on survival probability in our gastric cancer cohort. Gastric cancer patients carrying the rs10832417 GG genotype tended to have an inferior OS, compared with those carrying the rs10832417 GT or TT genotype (*P* = 0.009) (***Fig. 2A***). We further divided the participants into GG and GT + TT genotype subgroups and found that patients with the GT or TT genotype had a longer survival time, compared with rs10832417 GG genotype carriers (*P* = 0.003) (***Fig. 2B***). Because of the much smaller number of individuals in the TT genotype subgroup compared with the GG + TT genotype subgroup, there was no survival difference between these two subgroups (data not shown). Moreover, the effect of rs10832417 on gastric cancer survival was evaluated by stratified analysis under the dominant model. After adjustment for age, sex, smoking status, and drinking status, the T allele of rs10832417 was found to be a protective factor for gastric cancer prognosis in the subgroups of larger tumor size, T4 invasion, with lymph node metastasis, no distant metastasis, TNM stage Ⅲ, noncardia site, and intestinal histological type (*P* < 0.05) (***Table 3***). Overall, these results highlight that rs10832417 variants have prognostic potential in gastric cancer patients.

**Table 3:** Stratified analysis of the associations between rs10832417 genotypes and the OS of gastric cancer patients

Variables	Genotype (patients)		HR (95% CI)^a^	*P* ^a^	*P*_het_^b^
	GG	GT/TT			
Total	581	551	0.76 (0.63, 0.91)	**0.004**	
Tumor size					0.466
≤5 cm	358	357	0.82 (0.63, 1.06)	0.124	
>5 cm	223	194	0.71 (0.53, 0.94)	**0.017**	
Depth of invasion					0.936
T1	76	67	0.53 (0.17, 1.60)	0.256	
T2	82	77	0.73 (0.38, 1.41)	0.350	
T3	192	205	0.77 (0.56, 1.06)	0.101	
T4	230	202	0.76 (0.58, 0.99)	**0.040**	
Lymph node metastasis					0.933
N0	209	192	0.74 (0.48, 1.15)	0.176	
N1/N2/N3	372	359	0.76 (0.61, 0.93)	**0.009**	
Distant metastasis					0.246
M0	515	509	0.76 (0.62, 0.94)	**0.013**	
M1	66	42	1.03 (0.65, 1.65)	0.888	
TNM stage					0.889
Ⅰ	116	103	0.67 (0.31, 1.48)	0.322	
Ⅱ	97	112	0.92 (0.52, 1.64)	0.775	
Ⅲ	198	199	0.72 (0.53, 1.00)	**0.047**	
Ⅳ	169	137	0.78 (0.59, 1.04)	0.087	
Tumor site					0.953
Cardia	199	194	0.76 (0.56, 1.05)	0.093	
Non-cardia	382	357	0.76 (0.59, 0.96)	**0.022**	
Lauren type					0.370
Intestinal	446	408	0.71 (0.56, 0.90)	**0.004**	
Diffuse	125	129	0.86 (0.61, 1.20)	0.370	
^a^Adjusted for age, sex, smoking, and drinking status.^b^*P*-value for heterogeneity test.Abbreviations: T, tumor; N, lymph node; M, metastasis; HR, hazards ratio; CI, confidence interval.					

### Clinical application of rs10832417 in gastric cancer survival prediction

Univariable and multivariable Cox regression models were used to further determine whether rs10832417 is an independent prognostic predictor of survival of patients with gastric cancer in the presence of other demographic and clinicopathological variables. Results of the univariable analysis revealed that age, drinking habits, tumor size, depth of invasion, lymph node metastasis, distant metastasis, Lauren type, and rs10832417 genotypes were significantly associated with gastric cancer OS. The multivariable analysis, based on only the significant regression coefficients from the above-mentioned univariable results, indicated that all these variables remained statistically significant (*P* < 0.05; ***Supplementary Table 3***, available online).

**Figure 3:**
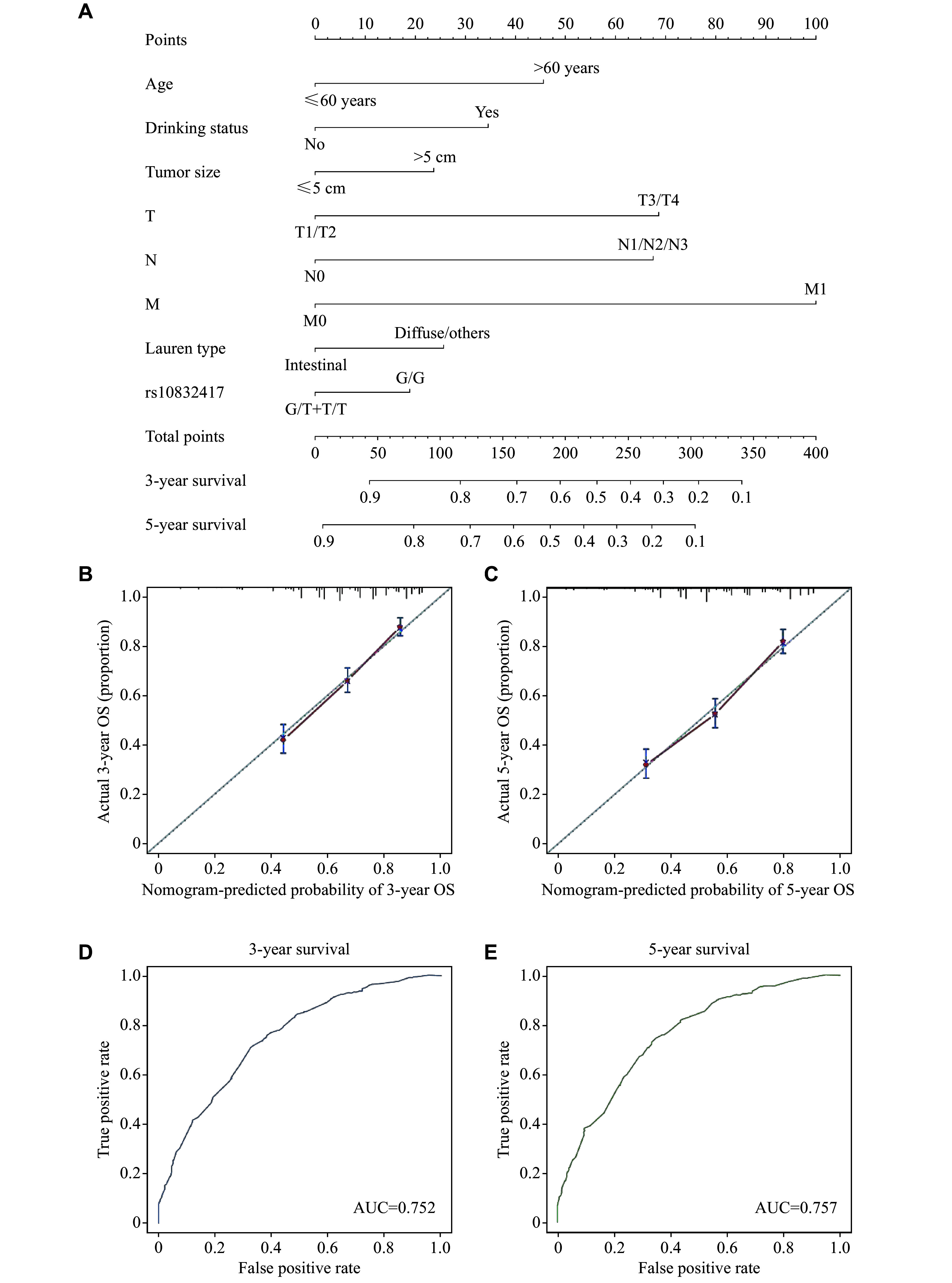
Gastric cancer survival nomogram and corresponding calibration curves. A: Nomogram for OS prediction of gastric cancer patients. B and C: Calibration curves of the nomogram in predicting 3- (B) and 5-year (C) OS in the in-house cohort. D and E: ROC curves of the nomogram in predicting 3- (D) and 5-year (E) OS in the in-house cohort. Abbreviations: T, tumor; N, lymph node; M, metastasis; OS, overall survival; AUC, area under the curve.

In light of clinical and translational focus of the present study, we next established a nomogram integrating rs10832417 genotypes and survival-associated factors to predict the survival probability of patients with gastric cancer. As shown in ***Fig. 3A***, a higher total point value obtained by summing the scores of each assigned parameter in the nomogram was related to inferior 3- and 5-year OS rates. In addition, the C-index values of each variable included in the nomogram and the nomogram itself were calculated, which presented the most robust predictive value for gastric cancer survival, with a C-index of 0.71 (***Supplementary Table 4***, available online). Furthermore, the bootstrapped calibration plots visualized the excellent goodness-of-fit of the model for 3- and 5-year OS prediction (***Fig. 3B*** and ***3C***). The ROC curves verified the good performance of the model for survival prediction (***Fig. 3D*** and ***3E***). In summary, the findings further indicate the clinical significance of rs10832417 in predicting prognostic outcomes of gastric cancer patients.

### *In silico* functional annotation of rs10832417

We further investigated potential biological functions of rs10832417 using several web servers, mainly based on SNPinfo, RegulomeDB, and HaploReg v4.1. Although no biological effects of rs10832417 on transcription factor binding sites were found, the score of rs10832417 from RegulomeDB was up to 3a, and the annotation results from HaploReg v4.1 indicated its functions in motif changes (***Supplementary Table 5***, available online). In addition, the changes in RNA secondary structure caused by rs10832417G>T were algorithmically predicted by RNAfold (***Supplementary Fig. 3A*** and ***3B***, available online), with an increase in the minimum free energy from −8.20 to −5.40 kcal/mol. Furthermore, we depicted their changes in the minimum free energy structure, the thermodynamic ensemble of RNA structures, and the centroid structure in the form of mountain plots (***Supplementary Fig. 3C*** and ***3D***, available online).

### Association between *KCNQ1* and gastric cancer survival

The rs10832417 variant is located in the intronic region of *KCNQ1*, which belongs to *KCNQ* family genes encoding Kv channels. Interaction networks of *KCNQ1* were investigated through STRING, and the results showed that *KCNQ1* interacted with some voltage-gated ionic channels (*i.e.*, *KCNE1-4*, *KCNE1L*, and *KCNJ2*) and their regulators (*i.e.*, *CALM1-3* and *AKAP9*) (***Supplementary Fig. 4A***, available online), which highlight the critical role of *KCNQ1* in ionic mechanisms. Then, we analyzed the expression profile of *KCNQ1* in gastric cancer as well as pan-cancers through various public datasets. There were no significant differential expression levels of *KCNQ1* between gastric cancer and adjacent normal tissues (***Supplementary Fig. 4B***, available online) or between gastric cancer and normal stomach samples, including adjacent normal tissues (***Supplementary Fig. 4C***, available online). However, differential expression levels of *KCNQ1* were observed among different molecular subtypes of gastric cancer (*P* < 0.05), with relatively high expression levels in the HM-indel subgroup (***Supplementary Fig. 4D***, available online). In addition, the expression patterns of *KCNQ1* lacked tissue specificity and did not demonstrate a similar expression tendency among pan-cancers and their corresponding adjacent normal tissues (***Supplementary Fig. 4E***, available online). However, the expression levels of *KCNQ1* were higher in stomach samples than in the majority of other GTEx samples (***Supplementary Fig. 4F***, available online).

To better understand the survival effects of *KCNQ1* in gastric cancer, we used datasets from TCGA-STAD and GEO to assess the survival probability related to different *KCNQ1* expression levels. The results showed that higher expression levels of *KCNQ1* were associated with higher OS and disease-free survival rates in TCGA-STAD divided by its median expression level (*P* < 0.05) (***Supplementary Fig. 5A*** and ***5B***, available online). We subsequently estimated the prognostic performance of *KCNQ1* in meta-datasets from GEO, and consistent results were observed for OS, first progression, and post-progression survival analyses of gastric cancer survival divided by the best cutoff value of its expression levels (***Supplementary Fig. 6***, available online). Collectively, *KCNQ1* showed its high progression- and survival-associated potential in gastric cancer based on the data from multiple public datasets.

### Mutation status and immune infiltration estimation of *KCNQ1* in gastric cancer

Genomic studies have identified the prognostic or predictive significance of a host of mutations in gastric cancer. Hence, we used this strategy to investigate the effect of the mutation status of *KCNQ1* on its expression in gastric cancer. The mutation rate of *KCNQ1* in gastric cancer was approximately among the average, compared with that in pan-cancers (***Supplementary Fig. 7A***, available online). Because the mutation rates of *KCNQ1* in various public datasets were no less than 1% (***Supplementary Fig. 7B***, available online), *KCNQ1* mutation may not have a major influence on its expression in gastric cancer (***Supplementary Fig. 7C***, available online). Therefore, future studies on the mutation functions of *KCNQ1* in gastric cancer are needed.

Moreover, the correlation between *KCNQ1* expression levels and immune infiltration levels in gastric cancer was analyzed and visualized based on the XCELL and TIMER methods^[19–20]^. As shown in ***Supplementary Fig. 7D*** (available online), *KCNQ1* expression was significantly associated with infiltration of various myeloid, lymphoid, stem and stromal cells, suggesting its potential roles in immunotherapy for gastric cancer.

## Discussion

Previous investigators have identified some critical roles of *KCNQ* family genes in cancer risk, progression, and treatment because of their ionic mechanisms^[6]^. However, the associations between genetic variants of *KCNQ* family genes and gastric cancer survival have received limited attention. In the present study, a large Chinese patient cohort was used to systematically evaluate the effects of candidate SNPs of *KCNQ* family genes on the survival of patients with gastric cancer. The results showed that rs10832417G>T in *KCNQ1* was associated with improved gastric cancer survival in the whole cohort as well as in some clinical subgroups. Moreover, a nomogram was established based on a combination of rs10832417 variants and other clinical factors for robust prognostic prediction and easier clinical translation in gastric cancer.

KCNQ1 has been reported as a prominent member of voltage-gated ion channels, which are widely distributed from the nervous system to the gastrointestinal tract^[21]^. *KCNQ1* has also been demonstrated to act as a tumor suppressor gene in some gastrointestinal cancers. For example, the downregulation of *KCNQ1* expression has been reported to be associated with an inferior survival in colon cancer, and may guide the decision on adjuvant chemotherapy in patients with stage Ⅱ microsatellite stable colon cancer^[22]^. High KCNQ1 expression detected by immunohistochemical staining was found to be associated with the improved OS in a cohort of colorectal cancer patients with liver metastasis^[23]^. However, the *KCNQ1* deficiency was observed in hepatocellular carcinoma cells and tissue, compared with the corresponding normal tissue, and it was identified as a prognostic predictor for a poor survival in hepatocellular carcinoma^[24]^. In the present study, based on multiple public datasets, we did not find significant differential expression of *KCNQ1* between gastric cancer and normal tissues, but prognostic significance existed between gastric cancer patients dichotomized by high- and low-expression levels of *KCNQ1*, indicating the possible effects of posttranscriptional processing of *KCNQ1* during gastric cancer progression. Hence, further *in vivo* and *in vitro* experiments are needed to investigate the underlying molecular mechanisms.

Advanced genome sequencing technologies have allowed for an easier assessment of genomic information in diseases, such as SNPs that may be associated with disease susceptibility and outcomes. Many studies have revealed the associations of some SNPs in *KCNQ1* with type 2 diabetes susceptibility^[25–26]^, cardiovascular disorders^[27]^, gout arthritis^[28]^, pancreatic cancer^[29]^, and others. For example, a retrospective study identified *KCNQ1* rs163182 as an independent predictor of treatment response in gastric cancer patients who received first-line EOF chemotherapy (epirubicin, oxaliplatin, and 5-fluorouracil), but it failed to find significant associations of rs163182 variants with OS or progression-free survival of patients with gastric cancer^[30]^. Similarly, *KCNQ1* rs231348 was reported to be associated with gastric cancer risk in another case-control study but not with gastric cancer survival^[31]^. A large number of participants in the two published studies had early-stage disease, which might contribute to the differences in their survival analysis.

Notably, the loss/gain-of-function mutations have been identified as pathophysiological drivers in various diseases. Hundreds of mutations located in *KCNQ1* have been reported to be associated with cardiac electrical activity, including atrial fibrillation, long QT syndrome, and short QT syndrome, which are common causes of lethal cardiac arrhythmias and subsequent sudden death^[32–34]^. Meanwhile, the mutational landscape of gastric cancer has revealed the roles of mutations in prognosis and treatment, such as mutations in *TP53*, *ARID1A*, *BRCA2*, and *CDH1*^[35–36]^. Although no expression difference was found between wild-type and mutant *KCNQ1* in gastric cancer in some public datasets, the biological functions underlying *KCNQ1* mutation need more exploration.

There are some limitations to the present study. We deliberately assessed a block of SNPs located in *KCNQ1*; however, advances in high-throughput technologies may allow for more comprehensive genomic discoveries regarding the associations between genetic variants in *KCNQ1* and gastric cancer survival. In addition, a large prospectively recruited study should be conducted on the survival of gastric cancer patients from multiple medical centers. Additionally, the functional results of the present study are only based on *in silico* prediction. Therefore, further biological experiments are needed to identify the potential molecular mechanisms responsible for the survival effects of *KCNQ1* and rs10832417 in gastric cancer.

## SUPPLEMENTARY DATA

Supplementary data to this article can be found online.

## References

[1] (2020). Gastric cancer. Lancet.

[2] (2021). Global cancer statistics 2020: GLOBOCAN estimates of incidence and mortality worldwide for 36 cancers in 185 countries. CA Cancer J Clin.

[3] (2018). Epigenetic biomarkers in gastrointestinal cancers: The current state and clinical perspectives. Semin Cancer Biol.

[4] (2017). Gastric adenocarcinoma. Nat Rev Dis Primers.

[5] (2018). KCNQ-encoded potassium channels as therapeutic targets. Annu Rev Pharmacol Toxicol.

[6] (2020). Kv7 channels in lung diseases. Front Physiol.

[7] (2000). Neuronal KCNQ potassium channels: physiology and role in disease. Nat Rev Neurosci.

[8] (2001). KCNQ potassium channels: physiology, pathophysiology, and pharmacology. Pharmacol Ther.

[9] (2020). The miR-140-5p/KLF9/KCNQ1 axis promotes the progression of renal cell carcinoma. FASEB J.

[b10] 10Ling J, Erol F, Viatchenko-Karpinski V, et al. Orofacial neuropathic pain induced by oxaliplatin: downregulation of KCNQ2 channels in V2 trigeminal ganglion neurons and treatment by the KCNQ2 channel potentiator retigabine[J]. Mol Pain, 2017, 13: 1744806917724715.

[11] (2011). Novel role of KCNQ2/3 channels in regulating neuronal cell viability. Cell Death Differ.

[12] (2022). Associations between *KCNQ1* and *ITIH4* gene polymorphisms and infant weight gain in early life. Pediatr Res.

[13] (2015). Variant rs2237892 of *KCNQ1* is potentially associated with hypertension and macrovascular complications in type 2 diabetes mellitus in a Chinese Han population. Genomics Proteomics Bioinformatics.

[14] (2014). Application of a combination of a knowledge-based algorithm and 2-stage screening to hypothesis-free genomic data on irinotecan-treated patients for identification of a candidate single nucleotide polymorphism related to an adverse effect. PLoS One.

[15] (2006). The contribution of genes involved in potassium-recycling in the inner ear to noise-induced hearing loss. Hum Mutat.

[16] (2009). Analysis of gene polymorphisms associated with K^+^ ion circulation in the inner ear of patients susceptible and resistant to noise-induced hearing loss. Ann Hum Genet.

[17] (2020). IKBKB rs2272736 is associated with gastric cancer survival. Pharmgenomics Pers Med.

[18] (2011). Clinical significance of the expression of DNA methyltransferase proteins in gastric cancer. Mol Med Rep.

[19] (2017). xCell: digitally portraying the tissue cellular heterogeneity landscape. Genome Biol.

[20] (2020). TIMER2.0 for analysis of tumor-infiltrating immune cells. Nucleic Acids Res.

[21] (2021). Hormonal signaling actions on Kv7.1 (KCNQ1) channels. Annu Rev Pharmacol Toxicol.

[22] (2016). Loss of KCNQ1 expression in stage II and stage III colon cancer is a strong prognostic factor for disease recurrence. Br J Cancer.

[23] (2014). The role of *KCNQ1* in mouse and human gastrointestinal cancers. Oncogene.

[24] (2018). Hypermethylated KCNQ1 acts as a tumor suppressor in hepatocellular carcinoma. Biochem Biophys Res Commun.

[25] (2015). Genetic fine mapping and genomic annotation defines causal mechanisms at type 2 diabetes susceptibility loci. Nat Genet.

[26] (2021). Integrated analysis of probability of type 2 diabetes mellitus with polymorphisms and methylation of *KCNQ1* gene: a nested case-control study. J Diabetes.

[27] (2021). Genetic variants associated with inherited cardiovascular disorders among 13,131 asymptomatic older adults of European descent. npj Genom Med.

[28] (2015). Genome-wide association analysis identifies three new risk loci for gout arthritis in Han Chinese. Nat Commun.

[29] (2014). Case-control study of diabetes-related genetic variants and pancreatic cancer risk in Japan. World J Gastroenterol.

[30] (2015). Effects of *IGF2BP2, KCNQ1* and *GCKR* polymorphisms on clinical outcome in metastatic gastric cancer treated with EOF regimen. Pharmacogenomics.

[31] (2021). Association study between *KCNQ1* and *KCNQ1OT1* genetic polymorphisms and gastric cancer susceptibility and survival in a Chinese Han population: a case-control study. Ann Transl Med.

[32] (2003). KCNQ1 gain-of-function mutation in familial atrial fibrillation. Science.

[33] (2008). Long QT syndrome. Curr Probl Cardiol.

[34] (2018). Mechanisms of KCNQ1 channel dysfunction in long QT syndrome involving voltage sensor domain mutations. Sci Adv.

[35] (2015). Mutational landscape of gastric adenocarcinoma in Chinese: implications for prognosis and therapy. Proc Natl Acad Sci U S A.

[36] (2020). Hereditary diffuse gastric cancer: updated clinical practice guidelines. Lancet Oncol.

