# Supplementary material for: Identification of common genetic variants in KCNQ family genes associated with gastric cancer survival in a Chinese population
Source: J Biomed Res. 2024 May 29;39(1):76–86. doi: 10.7555/JBR.38.20240040 (PMC11873595; doi:10.7555/JBR.38.20240040)
Supplement: Supplementary file 1 — Supplementary data to this article can be found online. [file jbr-39-1-76-S1.pdf]

Available online at www.jbr-pub.org.cn

Open Access at PubMed Central

Journal of Biomedical Research, 2025 39(1): 1–8

Supplementary Data

JBR

Identification of common genetic variants in *KCNQ* family genes associated with gastric cancer survival in a Chinese population

Yuetong Chen[1,2,△], Chen Li[2,△], Yi Shi[3,△], Jiali Dai[4], Yixuan Meng[5,6], Shuwei Li[5,6], Cuiju Tang[2], Dongying Gu[2,✉], Jinfei Chen[3,✉]

[1]*Department of Radiation Oncology, the Affiliated Suzhou Hospital of Nanjing Medical University, Suzhou Municipal Hospital, Gusu School, Nanjing Medical University, Suzhou, Jiangsu 215008, China;*
[2]*Department of Oncology, Nanjing First Hospital, Nanjing Medical University, Nanjing, Jiangsu 210006, China;*
[3]*Department of Oncology, the First Affiliated Hospital of Wenzhou Medical University, Wenzhou, Zhejiang 325015, China;*
[4]*Department of Oncology, the First Affiliated Hospital of Nanjing Medical University, Nanjing, Jiangsu 210029, China;*
[5]*Department of Environmental Genomics, Jiangsu Key Laboratory of Cancer Biomarkers, Prevention and Treatment, Collaborative Innovation Center for Cancer Personalized Medicine, Nanjing Medical University, Nanjing, Jiangsu 211166, China;*
[6]*Department of Genetic Toxicology, the Key Laboratory of Modern Toxicology of Ministry of Education, Center for Global Health, School of Public Health, Nanjing Medical University, Nanjing, Jiangsu 211166, China.*

Supplementary Table 1 **Clinicopathological characteristics of the gastric cancer cohort**

Variables	Patients (*n*=1 135)	Deaths (*n*=433)	HR (95% CI)	Log-rank *P*
Age (years)				<0.001
≤60	440	141	1.00	
>60	695	292	1.53 (1.25, 1.87)	
Sex				0.144
Male	824	326	1.00	
Female	311	107	0.85 (0.68, 1.06)	
Smoking status				0.240
No	945	364	1	
Yes	190	69	1.17 (0.90, 1.51)	
Drinking status				0.019
No	1 039	391	1.00	
Yes	96	42	1.46 (1.06, 2.01)	

△These authors contributed equally to this work.

✉Corresponding authors: Dongying Gu, Department of Oncology, Nanjing First Hospital, Nanjing Medical University, 68 Changle Road, Nanjing, Jiangsu 210006, China. E-mail: dygu@njmu.edu.cn; Jinfei Chen, Department of Oncology, the First Affiliated Hospital of Wenzhou Medical University, Nanbaixiang Street, Wenzhou, Zhejiang 325015, China. E-mail: jinfeichen@sohu.com.

Received: 18 February 2024; Revised: 26 April 2024; Accepted: 06 May 2024; Published online: 29 May 2024

CLC number: R735.2, Document code: A

The authors reported no conflict of interests.

This is an open access article under the Creative Commons Attribution (CC BY 4.0) license, which permits others to distribute, remix, adapt and build upon this work, for commercial use, provided the original work is properly cited.

© 2025 by Journal of Biomedical Research.

https://doi.org/10.7555/JBR.38.20240040

Supplementary Table 1 **Clinicopathological characteristics of the gastric cancer cohort** (Continued)

Variables	Patients (*n*=1 135)	Deaths (*n*=433)	HR (95% CI)	Log-rank *P*
Tumor size				<0.001
≤5cm	718	232	1.00	
>5 cm	417	201	1.81 (1.50, 2.19)	
Depth of invasion				<0.001
Tis	1	0		
T1	143	14	1.00	
T2	159	38	2.24 (1.22, 4.14)	
T3	398	153	4.61 (2.66, 7.96)	
T4	434	228	6.05 (3.53, 10.37)	
Lymph node metastasis				<0.001
N0	401	82	1.00	
N1/N2/N3	734	351	2.84 (2.23, 3.61)	
Distant metastasis				<0.001
M0	1 027	348	1.00	
M1	108	85	3.41 (2.68, 4.33)	
TNM stage				<0.001
Ⅰ	219	26	1.00	
Ⅱ	209	48	2.46 (1.52, 3.96)	
Ⅲ	398	155	3.84 (2.54, 5.82)	
Ⅳ	308	204	7.10 (4.72, 10.68)	
Tumor site				0.415
Cardia	394	160	1.00	
Non-cardia	741	273	0.92 (0.76, 1.12)	
Lauren type				< 0.001
Intestinal	857	289	1.00	
Diffuse	254	137	1.65 (1.34, 2.02)	
Others	24	7	1.02 (0.48, 2.15)	

The data was assessed by the Cox proportional-hazards model and log-rank test. Abbreviations: T, tumor; N, lymph node; M, metastasis; HR, hazards ratio; CI, confidence interval.

Supplementary Table 2 **Associations between genetic variants in *KCNQ1* and overall survival of gastric cancer patients**

Chr	SNP	Position (h19)	Ref/Alt allele	MAF	$P_{(HWE)}$	Call rate (%)	HR (95% CI)[a]	P^a
11	rs7108478	2628804	A/G	0.24	0.32	97.89	0.92 (0.78, 1.09)	0.339
11	rs4930137	2630594	T/C	0.30	0.29	95.42	0.93 (0.80, 1.08)	0.322
11	rs4930138	2630992	A/T	0.19	1.00	92.42	1.02 (0.86, 1.21)	0.843
11	rs7942590	2633715	C/G	0.19	1.00	98.50	1.00 (0.84, 1.18)	0.974
11	rs2106467	2635797	G/T	0.14	1.00	85.02	1.07 (0.88, 1.32)	0.494
11	**rs2106464**	2639233	C/T	0.11	0.70	99.38	0.75 (0.58, 0.96)	**0.022**
11	rs11023485	2639712	A/G	0.41	0.22	98.68	0.93 (0.81, 1.08)	0.357
11	rs2412058	2641129	A/G	0.24	0.32	74.36	0.97 (0.80, 1.17)	0.754
11	rs11023535	2648624	A/G	0.17	0.79	97.97	0.87 (0.72, 1.05)	0.149
11	rs79848425	2650021	C/G	0.10	0.23	86.43	0.81 (0.64, 1.04)	0.097

Supplementary Table 2 **Associations between genetic variants in *KCNQ1* and overall survival of gastric cancer patients** (Continued)

Chr	SNP	Position (h19)	Ref/Alt allele	MAF	$P_{(HWE)}$	Call rate (%)	HR (95% CI)[a]	P^a
11	**rs10832417**	2652657	G/T	0.38	1.00	99.74	0.84 (0.72, 0.98)	**0.023**
11	rs141541247	2665657	G/C	0.03	1.00	98.85	1.15 (0.83, 1.59)	0.408
11	rs12573965	2681632	A/C	0.05	1.00	98.94	1.18 (0.89, 1.56)	0.252
11	rs760419	2683357	A/G	0.40	1.00	99.91	0.87 (0.75, 1.01)	0.062
11	rs2075868	2683467	C/G	0.27	0.85	99.91	0.91 (0.78, 1.06)	0.243
11	rs12271234	2685947	T/C	0.20	1.00	96.92	0.92 (0.78, 1.08)	0.318
11	rs10832514	2688526	A/G	0.07	0.61	97.71	0.87 (0.70, 1.08)	0.199
11	rs16928527	2688705	T/A	0.11	0.14	99.30	0.93 (0.76, 1.14)	0.504
11	rs61870802	2692442	C/T	0.07	0.58	99.91	0.90 (0.69, 1.17)	0.419
11	rs60837062	2704155	T/C	0.04	1.00	96.12	1.14 (0.90, 1.44)	0.279
11	rs77639468	2706325	G/A	0.04	1.00	99.91	1.05 (0.76, 1.47)	0.759
11	rs7939976	2712286	A/G	0.08	1.00	99.21	0.91 (0.73, 1.14)	0.412
11	rs4930148	2717312	A/G	0.04	1.00	95.42	0.98 (0.73, 1.32)	0.903
11	rs463924	2717680	T/C	0.19	0.82	99.56	0.90 (0.76, 1.07)	0.235
11	rs78344341	2718313	T/G	0.11	0.48	93.04	0.85 (0.67, 1.08)	0.184
11	rs2283194	2722667	G/A	0.13	0.75	100.00	1.00 (0.81, 1.22)	0.966

[a]Adjusted for age, sex, smoking and drinking status in the additive models. Abbreviations: HWE, Hardy-Weinberg Equilibrium; MAF, minor allele frequency; HR, hazards ratio; CI, confidence interval.

Supplementary Table 3 **Selection of variables included in the nomogram by univariable and multivariable analysis**

Variables	Univariable analysis		Multivariable analysis	
	HR (95% CI)	*P*	HR (95% CI)	*P*
Age (>60 years *vs.* ≤60 years)	1.53 (1.25, 1.87)	<0.001	1.60 (1.31, 1.97)	<0.001
Sex (female *vs.* male)	0.85 (0.68, 1.06)	0.144		
Smoking status (yes *vs.* no)	1.17 (0.90, 1.51)	0.239		
Drinking status (yes *vs.* no)	1.46 (1.06, 2.01)	0.020	1.43 (1.04, 1.97)	0.028
Tumor size (>5 cm *vs.* ≤5 cm)	1.81 (1.50, 2.19)	<0.001	1.28 (1.05, 1.55)	0.014
T (T3/T4 *vs.* T1/T2)	3.20 (2.39, 4.27)	<0.001	2.04 (1.50, 2.77)	<0.001
N (N1/N2/N3 *vs.* N0)	2.84 (2.23, 3.61)	<0.001	2.01 (1.56, 2.60)	<0.001
M (M1 *vs.* M0)	3.41 (2.68, 4.33)	<0.001	2.82 (2.21, 3.61)	<0.001
Tumor site (non-cardia *vs.* cardia)	0.92 (0.76, 1.12)	0.414		
Lauren type (diffuse/others *vs.* intestinal)	1.60 (1.31, 1.95)	<0.001	1.31 (1.06, 1.60)	0.011
rs10832417 (GT/TT *vs.* GG)	0.75 (0.62, 0.91)	0.003	0.82 (0.68, 1.00)	0.046

Abbreviations: T, tumor; N, lymph node; M, metastasis; HR, hazards ratio; CI, confidence interval.

Supplementary Table 4 **C-index for our nomogram and variables included**

Variables	C-index	SE
Age	0.55	0.01
Drinking status	0.52	0.01
Tumor size	0.58	0.01
T	0.60	0.01
N	0.61	0.01
M	0.57	0.01
Lauren type	0.55	0.01
rs10832417	0.53	0.01
Nomogram	0.71	0.01

Abbreviations: T, tumor; N, lymph node; M, metastasis; SE, standard error.

Supplementary Table 5 **Functional prediction of rs10832417**

SNP	Gene	SNPinfo TFBS	RegulomeDB score	HaploReg v4.1	
				Motifs changed	dbSNP function annotation
rs10832417	*KCNQ1*	–	3a	18 altered motifs	intronic

Abbreviation: SNP, single nucleotide polymorphism.

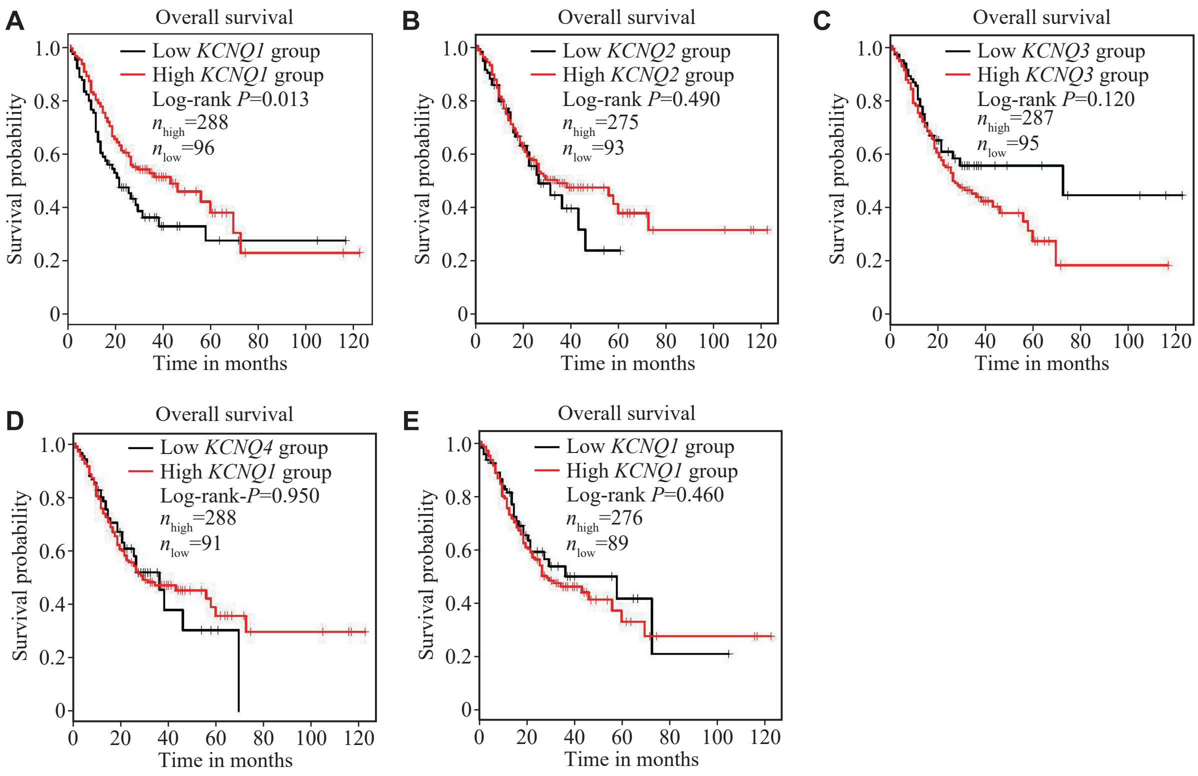

Supplementary Fig. 1 **Associations between *KCNQ* family genes and overall survival in gastric cancer.** A–E: The analyses were performed by the online tool GEPIA2 based on the TCGA-STAD dataset. Cox proportional-hazards model was applied and P < 0.05 was set as the cutoff value.

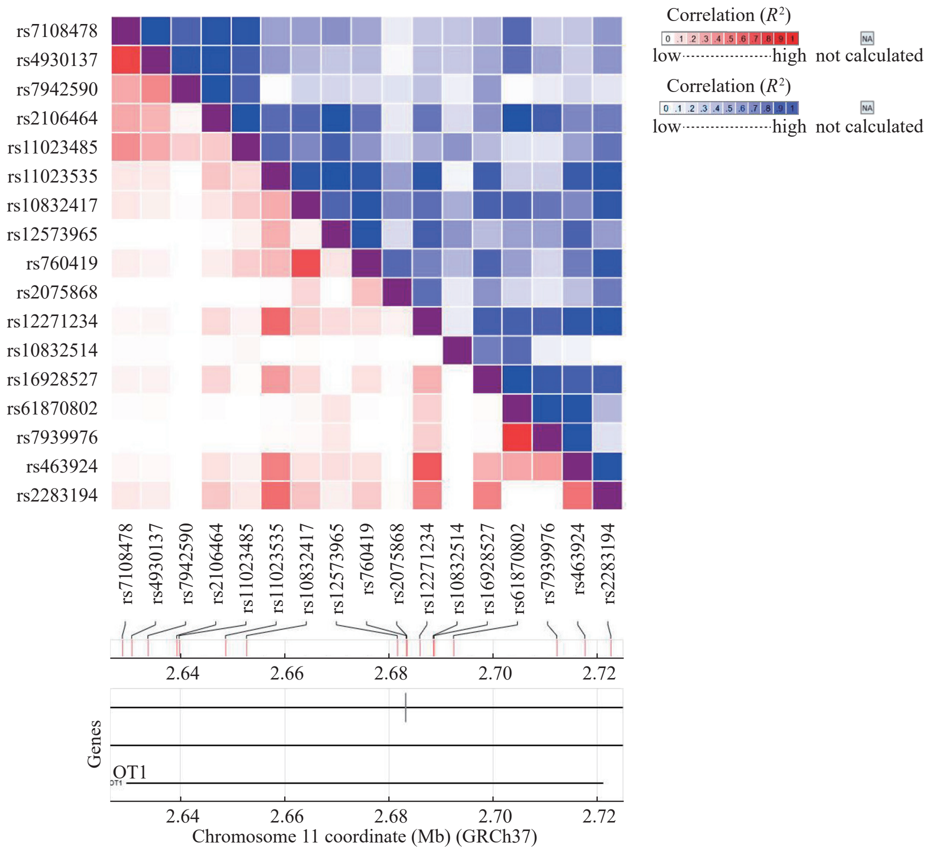

Supplementary Fig. 2 **Visualization of linkage disequilibrium among candidate single nucleotide polymorphisms calculated in the CHB and JBT populations.** Abbreviations: CHB, Han Chinese in Beijing; JPT, Japanese in Tokyo.

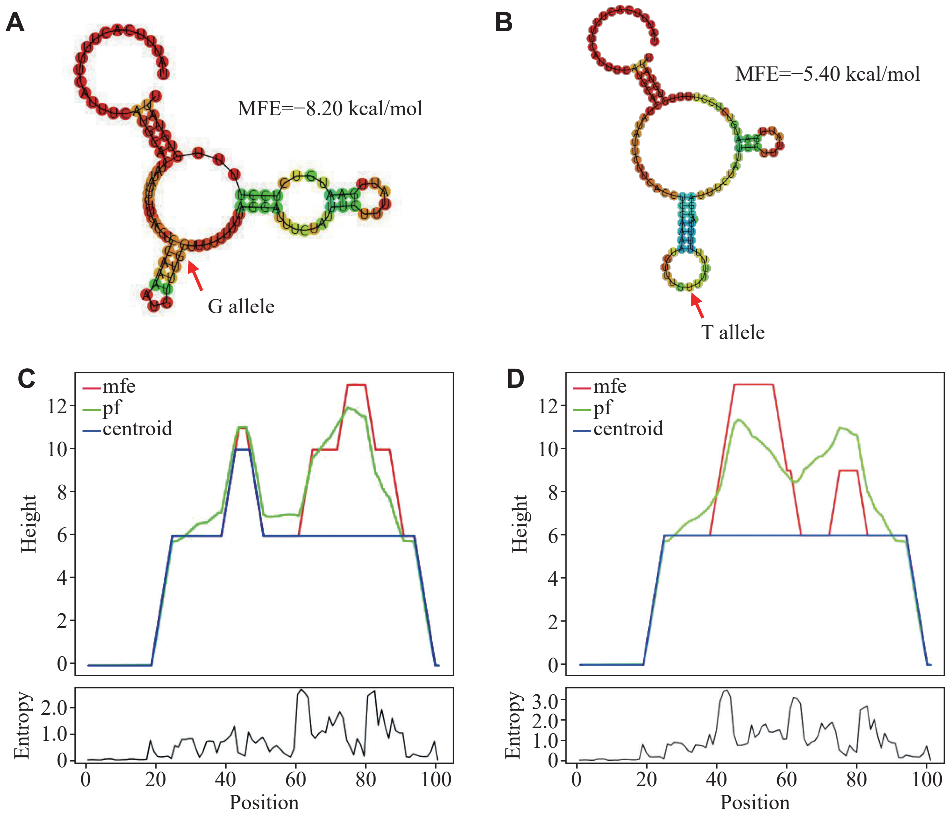

Supplementary Fig. 3 ***In silico*** **prediction of RNA secondary structure affected by rs10832417.** A and B: The MFE structures of rs10832417 with G and T alleles, respectively. C and D: The mountain plots representing the MFE structure, the thermodynamic ensemble of RNA structures, and the centroid structure of rs10832417 with G and T alleles. Abbreviations: MFE, minimum free energy; pf: partition function.

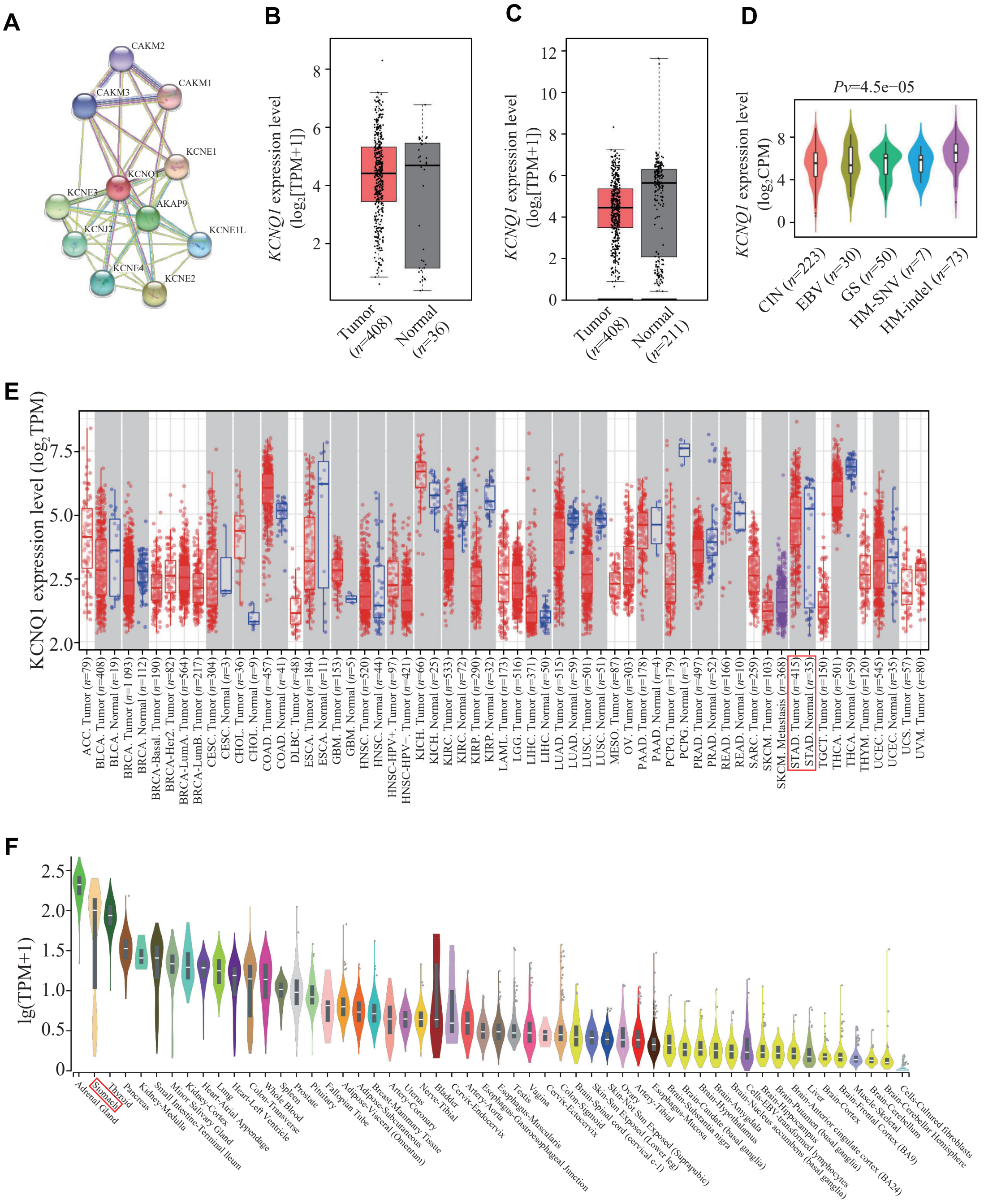

Supplementary Fig. 4 **Relative expression levels of *KCNQ1* in pan-cancers.** A: Interaction network of *KCNQ1* predicted by STRING web poral. B: Differential expression levels of *KCNQ1* between gastric cancer and normal tissues in the TCGA dataset. C: Differential expression levels of *KCNQ1* between gastric cancer and normal tissues in TCGA and GTEx datasets. D: Differential expression levels of *KCNQ1* among molecular subtypes of gastric cancer in TCGA. *Pv* means *P* value caculated by the Kruskal-Wallis test. E: Differential expression levels of *KCNQ1* between cancer and adjacent normal tissues across pan-cancers in TCGA. F: Relative expression levels of *KCNQ1* among different tissues in GTEx. Abbreviations: TCGA, The Cancer Genome Atlas; GTEx, Genotype-Tissue Expression. ACC, adrenocortical carcinoma; BLCA, bladder urothelial carcinoma; BRCA, breast invasive carcinoma; CESC, cervical squamous cell carcinoma and endocervical adenocarcinoma; CHOL, cholangiocarcinoma; COAD, colon adenocarcinoma; DLBC, lymphoid neoplasm diffuse large B-cell lymphoma; ESCA, esophageal carcinoma; GBM, glioblastoma multiforme; HNSC, head and neck squamous cell carcinoma; KICH, kidney chromophobe; KIRC, kidney renal clear cell carcinoma; KIRP, kidney renal papillary cell carcinoma; LAML, acute myeloid leukemia; LGG, brain lower grade glioma; LIHC, liver hepatocellular carcinoma; LUAD, lung adenocarcinoma; LUSC, lung squamous cell carcinoma; MESO, mesothelioma; OV, ovarian serous cystadenocarcinoma; PAAD, pancreatic adenocarcinoma; PCPG, pheochromocytoma and paraganglioma; PRAD, prostate adenocarcinoma; READ, rectum adenocarcinoma; SARC, sarcoma; SKCM, skin cutaneous melanoma; STAD, stomach adenocarcinoma; TGCT, testicular germ cell tumors; THCA, thyroid carcinoma; THYM, thymoma; UCEC, uterine corpus endometrial carcinoma; UCS, uterine carcinosarcoma; UVM, uveal melanoma; CIN, chromosomal-instable; EBV, Epstein-Barr-virus-positive; GS, genomically-stable; HM-SNV, hypermutated-single-nucleotide-variants; HM-Indel, hypermutated-insertion-deletion; TPM, transcripts per million; CPM, counts per million.

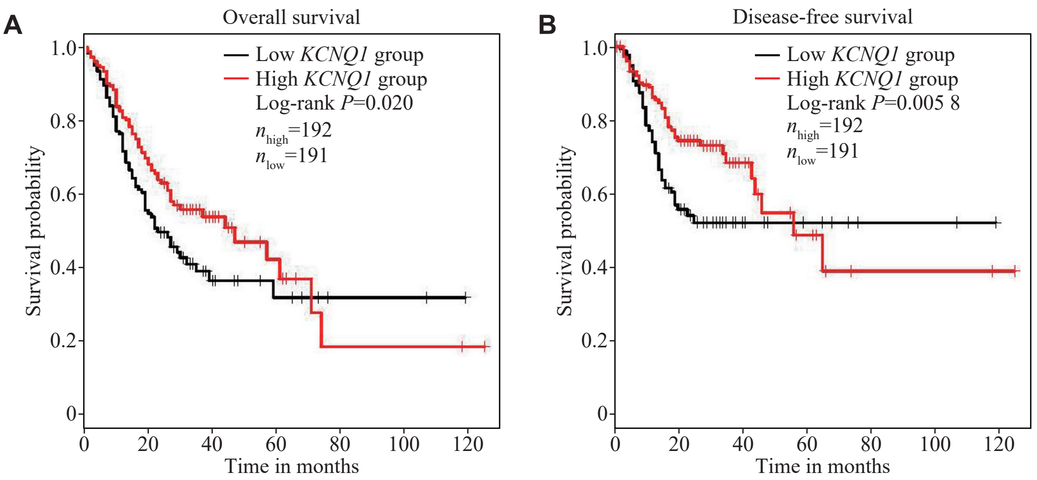

Supplementary Fig. 5 **Survival analysis of *KCNQ1* in gastric cancer.** Kaplan-Meier survival curve of *KCNQ1* for OS (A) and DFS (B) in gastric cancer. The analyses were performed by the online tool GEPIA2 based on the TCGA-STAD dataset. Cox proportional-hazards model was applied and $P < 0.05$ was set as the cutoff value. Abbreviations: OS, overall survival; DFS, disease free survival.

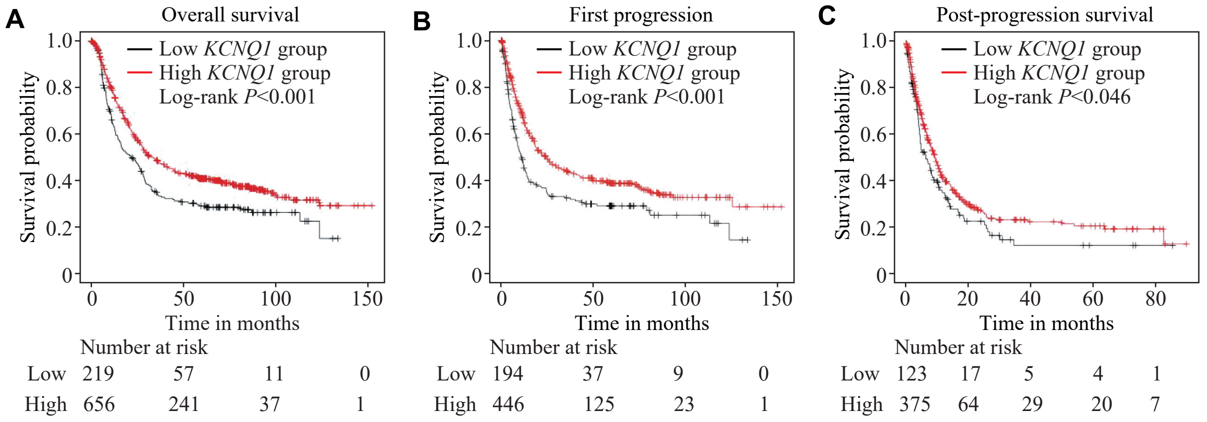

Supplementary Fig. 6 **Prognostic potential of *KCNQ1* in patients of gastric cancer.** Comparisons of overall survival (A), first progression (B), and post progression survival (C) between groups stratified by *KCNQ1* expression levels in the Kaplan Meier plotter web portal.

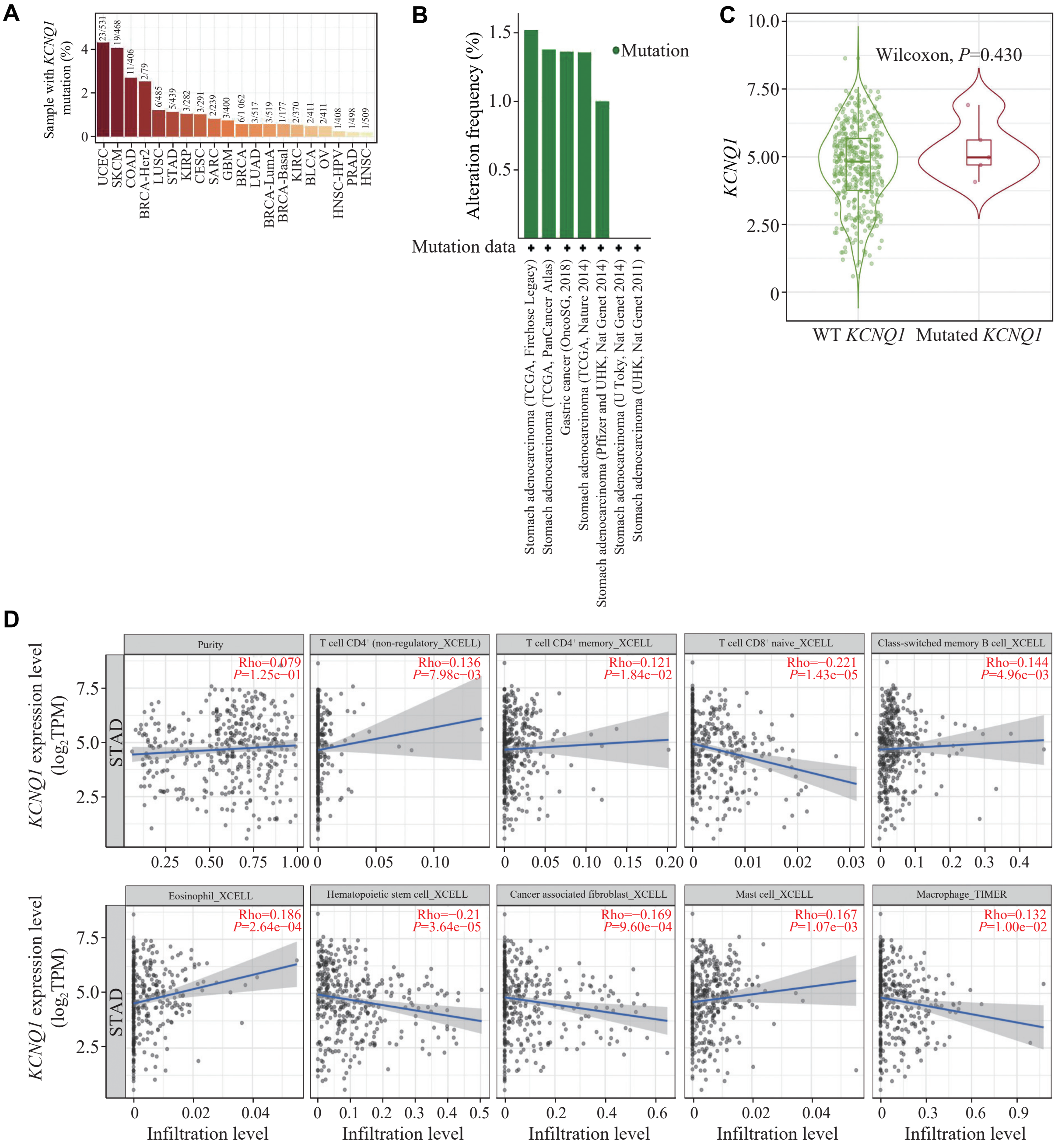

Supplementary Fig. 7 **Mutation frequency and immune infiltration estimation of *KCNQ1* in gastric cancer.** A: Mutation frequency of *KCNQ1* in diverse cancers. B: Mutation frequency of *KCNQ1* in gastric cancer estimated in multiple public datasets. The data were downloaded from the online tool TIMER 2.0 (http://timer.cistrome.org/), and the missed last two columns meant that those two datasets did not include the *KCNQ1* mutation data. C: Differential expression levels between wild-type (WT) and mutated *KCNQ1* in gastric cancer. D: Immune infiltration estimation of *KCNQ1* in gastric cancer. Abbreviations: UCEC, uterine corpus endometrial carcinoma; SKCM, skin cutaneous melanoma; COAD, colon adenocarcinoma; BRCA, breast invasive carcinoma; LUSC, lung squamous cell carcinoma; STAD, stomach adenocarcinoma; KIRP, kidney renal papillary cell carcinoma; CESC, cervical squamous cell carcinoma and endocervical adenocarcinoma; SARC, sarcoma; GBM, glioblastoma multiforme; LUAD, lung adenocarcinoma; KIRC, kidney renal clear cell carcinoma; BLCA, bladder urothelial carcinoma; OV, ovarian serous cystadenocarcinoma; HNSC, head and neck squamous cell carcinoma; PRAD, prostate adenocarcinoma; TPM, transcripts per million.
